# Supplementary material for: Electrical switching of high-performance bioinspired nanocellulose nanocomposites
Source: Nat Commun. 2021 Feb 26;12:1312. doi: 10.1038/s41467-021-21599-1 (PMC7910463; doi:10.1038/s41467-021-21599-1)
Supplement: Supplementary file 1 — Supplementary Information [file 41467_2021_21599_MOESM1_ESM.pdf]

Supplementary Information

Electrical Switching of High-Performance Bioinspired Nanocellulose Nanocomposites

Dejin Jiao[1,2,3], Francisco Lossada[1,2,3], Jiaqi Guo[1,2,3], Oliver Skarsetz[1,2,3], Daniel Hoenders[1,2,3,5], Jin Liu[1,2,3] and Andreas Walther[1,2,3,4,5]*

[1] Institute for Macromolecular Chemistry, Stefan-Meier-Str. 31, University of Freiburg, 79104 Freiburg, Germany.

[2] Freiburg Materials Research Center, Stefan-Meier-Str. 21, University of Freiburg, 79104 Freiburg, Germany.

[3] Freiburg Center for Interactive Materials and Bioinspired Technologies, Georges-Köhler-Allee 105, University of Freiburg, 79110 Freiburg, Germany.

[4] Cluster of Excellence *liv*MatS @ FIT – Freiburg Center for Interactive Materials and Bioinspired Technologies, University of Freiburg, Georges-Köhler-Allee 105, 79110 Freiburg, Germany

[5] Department of Chemistry, Duesbergweg 10-14, University of Mainz, 55128 Mainz, Germany

Correspondence and requests for materials should be addressed to A.W. (email: andreas.walther@uni-mainz.de)

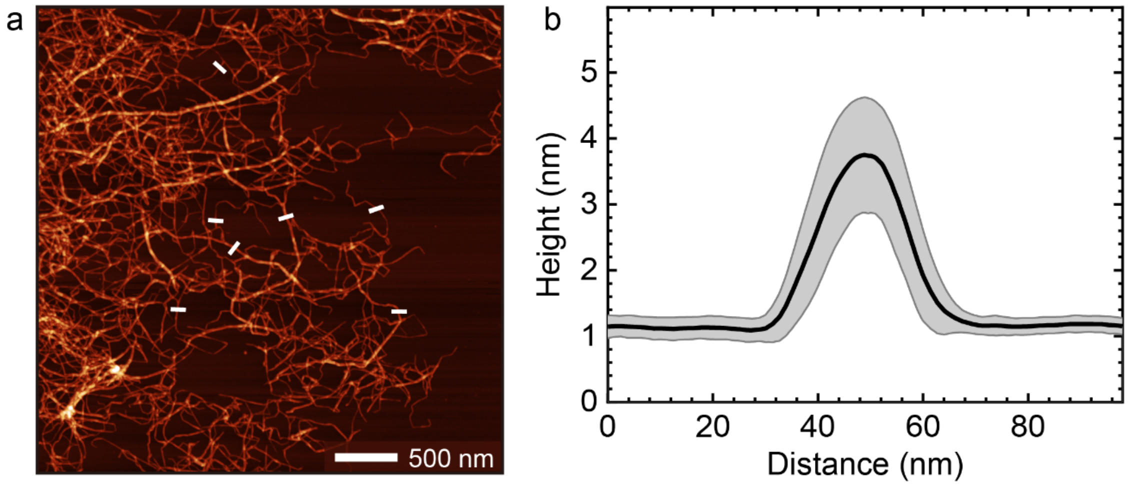

Supplementary Fig. 1. Atomic force microscopy (AFM) characterization of the diameters of CNFs. (a) AFM height image of CNFs. Z- scale is 12 nm. (b) Averages of 7 selected CNFs height profiles indicated in Figure (a). The average diameter is 2.6 ± 0.6 nm. The shaded area indicates the standard deviation of the selected height profiles.

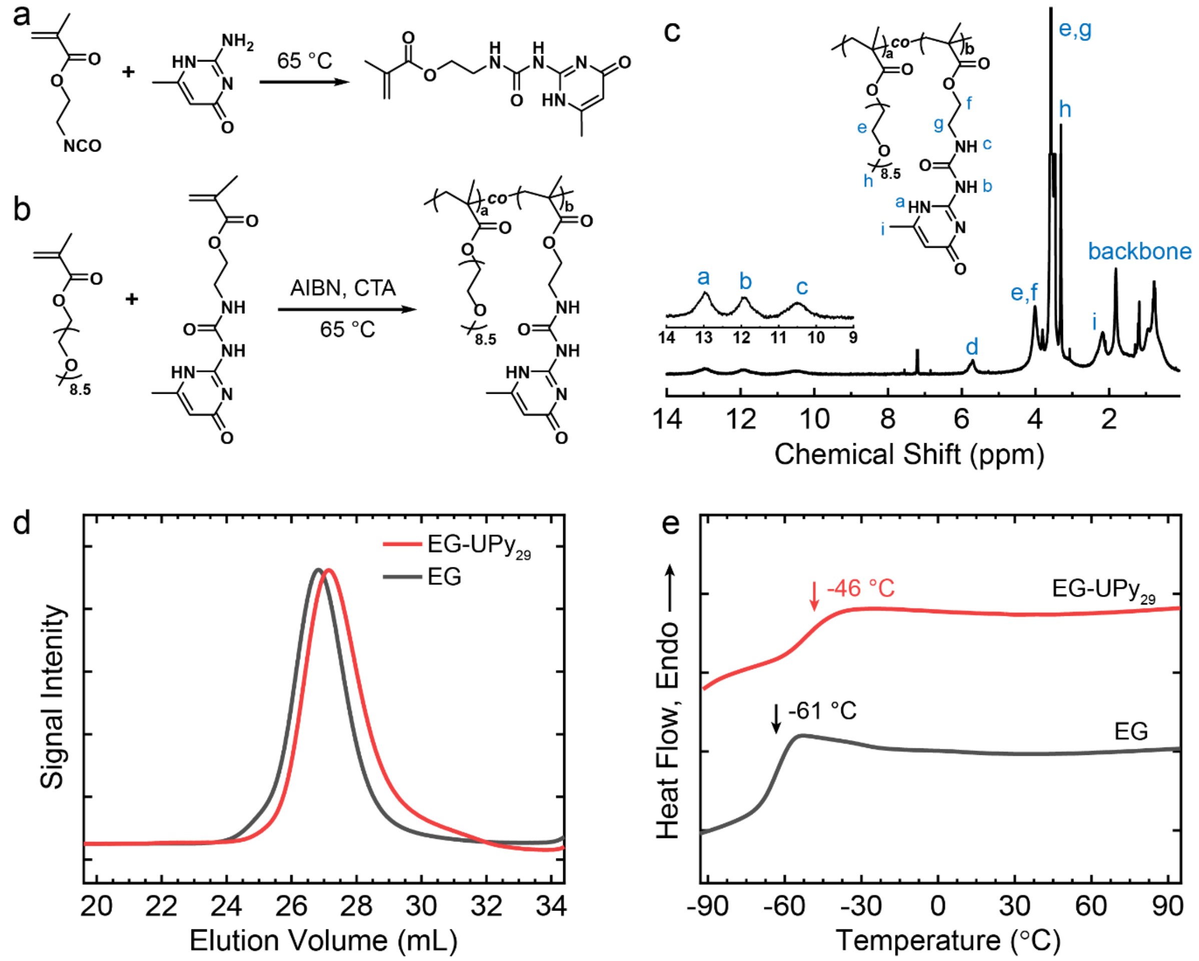

Supplementary Fig. 2. Synthesis of polymers using RAFT polymerization and copolymer properties. Schemes for (a) the synthesis of UPy monomer, UPyMA, (b) the RAFT copolymerization based on OEGMA and UPyMA. (c) ^{1}H NMR spectrum of EG-UPy$_{29}$ copolymer. (d) GPC traces of final EG-UPy$_{29}$ and EG polymers in DMAc. (e) DSC thermograms of EG-UPy$_{29}$ and EG polymers showing the T_gs.

Supplementary Table 1. Overview of polymers prepared by RAFT polymerization.

Sample	UPy units content (mol%)[a]	M_n (kDa)[b]	$Đ$[b]	T_g (°C)[c]
EG	0	51	1.12	-61
EG-UPy$_{29}$	29	40	1.12	-46

[a] Molar content of UPy motifs determined by ^{1}H NMR. [b] M_n and $Đ$ are measured by GPC in DMAc (corresponding GPC traces are in Figure S1d). [c] T_g is obtained from DSC (the thermograms are in Figure S2e).

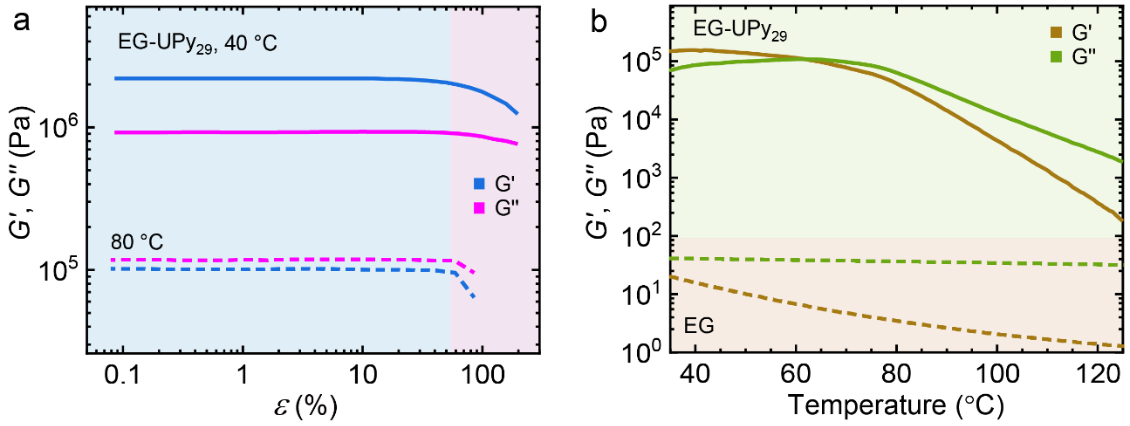

Supplementary Fig. 3. Rheological oscillation experiments. (a) Amplitude sweep of EG-UPy29 film at a frequency of 1 Hz. The linear viscoelastic regime is up to ca. ε = 50 %. (b) Temperature sweep (ε = 30 %, f = 1 Hz), heating EG-UPy29 and EG from 30 °C to 130 °C with a heating rate of 5 °C min^{-1}. The copolymer bearing thermo-reversible crosslinks, i.g. EG-UPy29 shows a thermal transition located at 62 °C, where the material behavior changes from elastomer (*G*'' < *G*') to melt-like behavior (*G*'' > *G*') with a drastic loss in *G*' across more than three orders of magnitude when going from room temperature to 130 °C. This corresponds to the thermal dynamization and dissociation of the hydrogen-bonded UPy dimers, and hence to the transition from a transient network to a polymer melt. By contrast, pure EG does not show any transition and behaves as viscous melt within the temperature range 30–130 °C. *G*' of EG only changes within one order of magnitude due to temperature-accelerated reptation and relaxation.

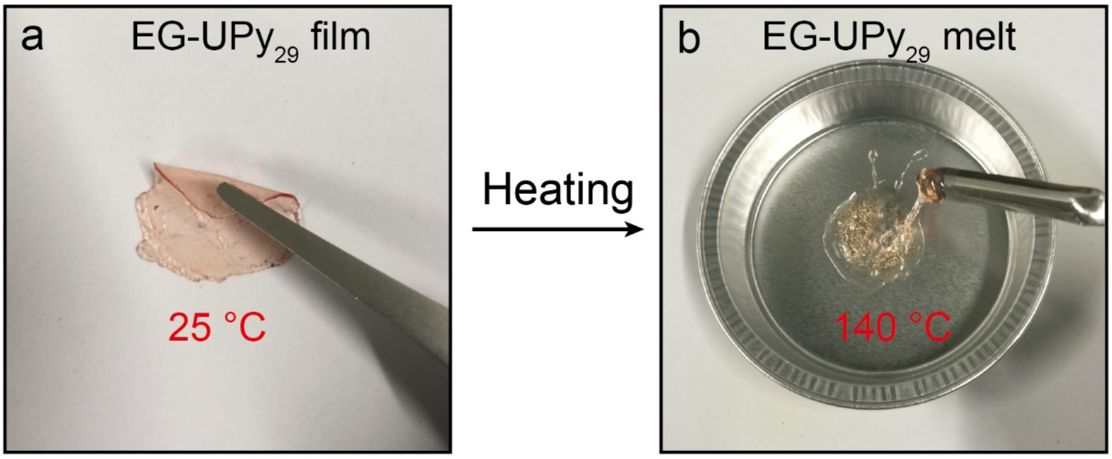

Supplementary Fig. 4. Solid-to-melt transition of EG-UPy_{29} polymer upon heating. (a) The polymer is an elastomer at 25 °C, (b) and turns to a melt at 140 °C

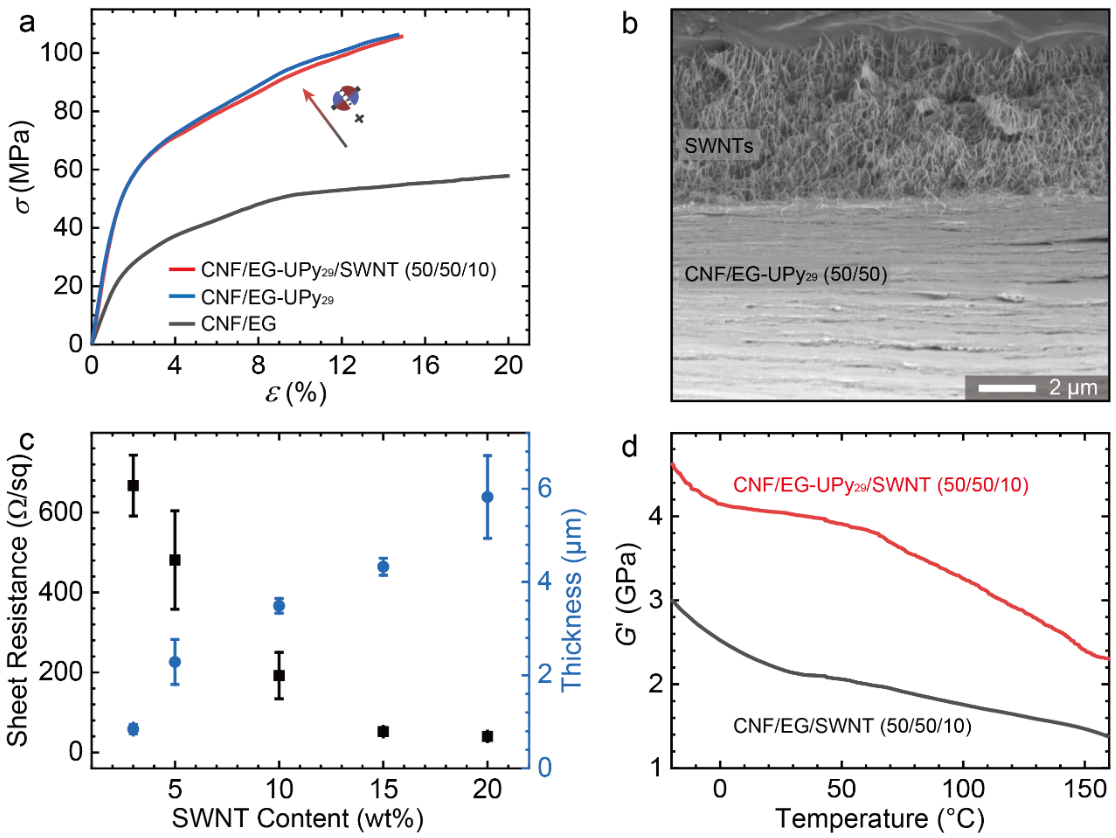

Supplementary Fig. 5. (a) Stress–strain curves for the CNF/EG and CNF/EG-UPy$_{29}$ nanocomposites with 50/50 CNF/polymer weight ratio, and the CNF/EG-UPy$_{29}$/SWNT (50/50/10) nanocomposite. (b) Cross-sectional SEM image for the CNF/EG-UPy$_{29}$/SWNT (50/50/10) nanocomposite. (c) Conductivity for CNF/EG-UPy$_{29}$/SWNT nanocomposites and thickness of conductive SWNT layers as a function of SWNT content. Error bars are standard deviations of duplicate measurements. (d) Storage modulus, *G'*, for CNF/EG/SWNT and CNF/EG-UPy$_{29}$/SWNT (50/50/10) nanocomposites.

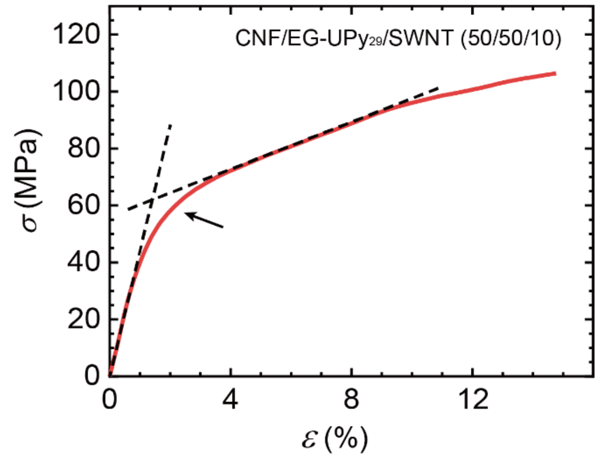

Supplementary Fig. 6. Determination of yield points. Stress–strain curves of CNF/EG-UPy_{29}/SWNT (50/50/10) nanocomposite. Yield points are determined as the intersection of the two linear regimes of the elastic and first plastic deformation zone.

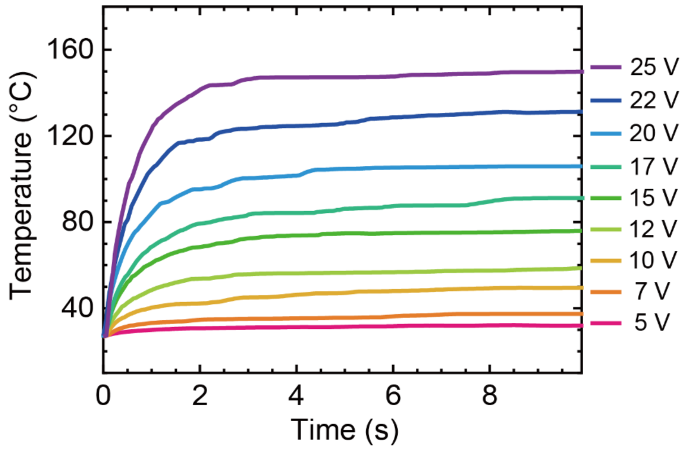

Supplementary Fig. 7. Time-dependent heating plot detected from the backside (without SWNT coating) for different applied Voltage.

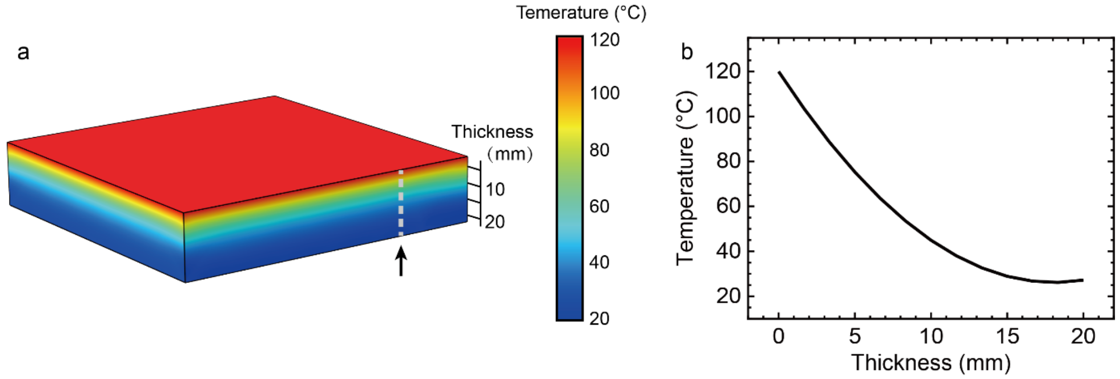

Supplementary Fig. 8. COMSOL finite element modeling (FEM) simulations for thermal conduction in a bulk material, in which the top layer is heated up to 120 °C. (a) The temperature map of the final state in the material. (b) The temperature gradient along the thickness, obtained from the dash line indicated in (a). The heat penetration is limited by the through-plane thermal conductivity of the material. The through-plane thermal conductivities of pure CNF nanopaper is 0.03 W/m·K, while thermal conductivities of most amorphous polymer range between 0.1-0.3 W/m·K.[1-3] In addition, the specific heat capacity (C_p) of CNFs nanopaper is 0.7 J/g·K, and C_p of most polymers is around 1.5 J/g·K at room temperature.[4] Assuming that the thermal conductivity and C_p of the material is 0.1 W/m·K and 1.0 J/g·K, respectively, it is possible to calculate the temperature changes along the thickness using FEM. As applied the EG-UPy system, it becomes clear that, when heated to 120 °C on top, the temperature is high enough for a UPy-UPy dissociation up to a thickness of ca. 7 mm.

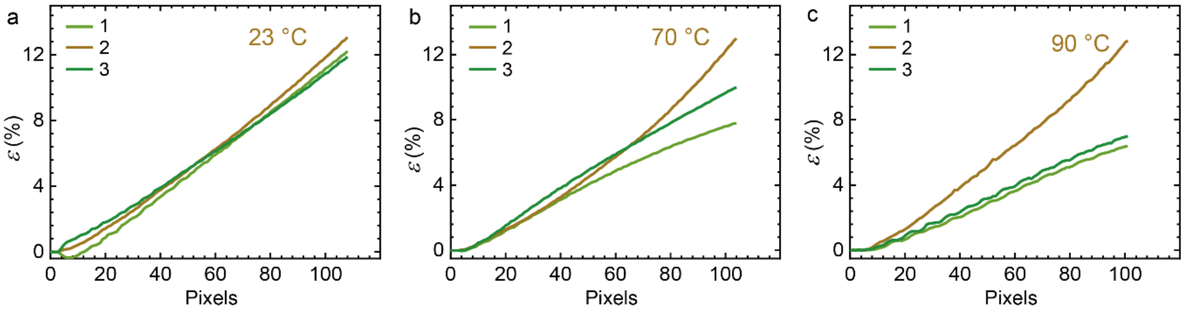

Supplementary Fig. 9. The true strain extracted from DIC at different local temperature in the middle using DIC. The lines 1-3 represent the parts 1-3 in Figure 4a. These temporal plots are acquired under Joule heating to (a) 23 °C. (b) 70 °C. (c) 90 °C.

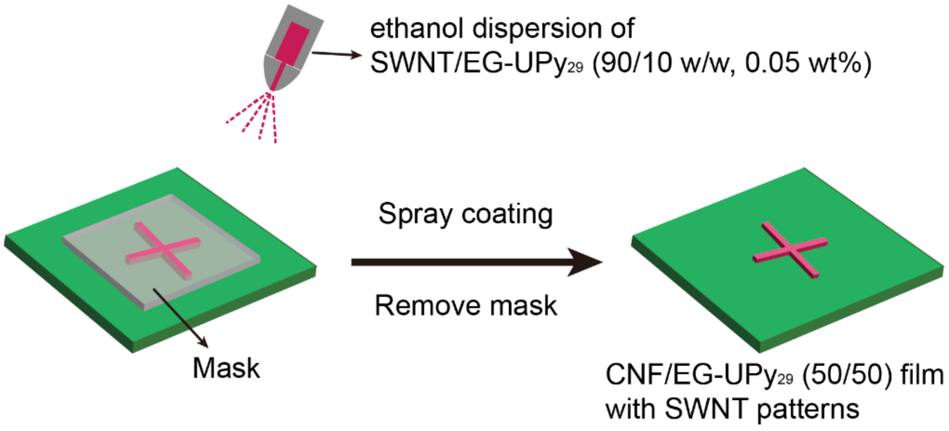

Supplementary Fig. 10. Schematic of direct patterning of SWNT thin layer by spray coating of SWNT-rich ethanol dispersion on the CNF/EG-UPy$_{29}$ (50/50) nanocomposites.

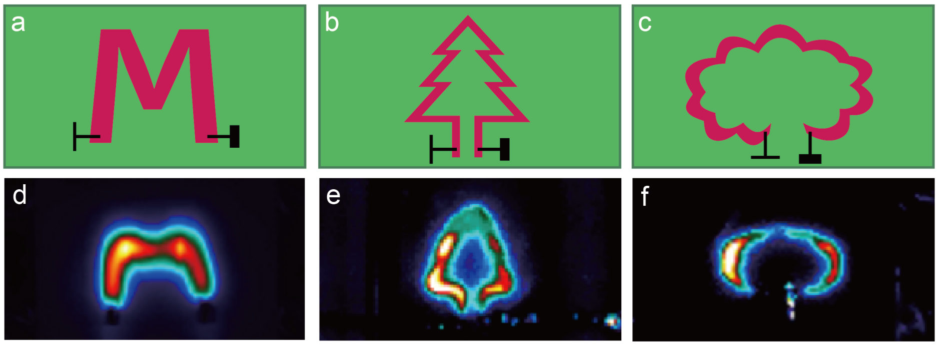

Supplementary Fig. 11. FLIR images showing the Joule heating patterns achieved by application of voltage on the SWNTs thin layers with predesigned patterns. (a-c) Schematic of SWNT thin layer with patterns on the CNF/EG-UPy_{29} (50/50) nanocomposites. (d-f) Corresponding FLIR images showing the patterns under applied voltage of 25 V.

References

1 Song, N. *et al.* Highly anisotropic thermal conductivity of layer-by-layer assembled nanofibrillated cellulose/graphene nanosheets hybrid films for thermal management. *ACS Appl. Mater. Interfaces* 9, 2924-2932 (2017).

2 Song, N. *et al.* Anisotropic thermally conductive flexible films based on nanofibrillated cellulose and aligned graphene nanosheets. *J. Mater. Chem. C* 4, 305-314 (2016).

3 Han, Z. & Fina, A. Thermal conductivity of carbon nanotubes and their polymer nanocomposites: A review. *Prog. Polym. Sci.* 36, 914-944 (2011).

4 Wen, J. in *Physical properties of polymers handbook* 145-154 (Springer, 2007).
