# Supplementary material for: Electrical switching of high-performance bioinspired nanocellulose nanocomposites
Source: Nat Commun. 2021 Feb 26;12:1312. doi: 10.1038/s41467-021-21599-1 (PMC7910463; doi:10.1038/s41467-021-21599-1)
Supplement: Supplementary file 3 — Description of Additional Supplementary Files [file 41467_2021_21599_MOESM3_ESM.pdf]

Description of Additional Supplementary Files

File Name: Supplementary Movie 1
Description: Electricity-Adaptive Mechanical Patterns

File Name: Supplementary Movie 2
Description: Reconfiguration of Mechanical Patterns
